# Case Report: Sequential multi-stage dermoscopic evolution of hypertrophic lichen planus in a patient with varicose veins

**DOI:** 10.3389/fmed.2026.1881656

**Published:** 2026-07-07

**Authors:** Xin Zhang, Junzhu Xu, Zhe Gao

**Affiliations:** Department of Dermatology, Hangzhou Third People's Hospital, Hangzhou, Zhejiang, China

**Keywords:** chronic venous insufficiency, corn pearls, dermoscopy, hypertrophic lichen planus, Wickham striae

## Abstract

Hypertrophic lichen planus (HLP) is a chronic, T-cell-mediated inflammatory dermatosis characterized by verrucous, hyperkeratotic papules and predominantly affecting the lower extremities. Dermoscopy has emerged as a valuable non-invasive tool for diagnosing HLP; however, sequential dermoscopic documentation spanning multiple evolutionary stages within a single patient has rarely been reported. Here, we describe a 62-year-old male who presented with progressively enlarging hyperkeratotic nodules on the right lower leg, notably concentrated on the side affected by varicose veins. Dermoscopic examination of lesions at different anatomical sites simultaneously revealed distinct sequential features corresponding to discrete stages of HLP evolution: (1) structureless pigmented areas with hairpin and dotted vessels; (2) diffuse pigmentation with emerging Wickham striae and keratinous plugs (corn pearls); (3) volcano crater-like morphology with elevated keratinous plugs and violaceous striae; and (4) fully developed “bouquet of white roses” pattern with lobulated hyperkeratotic masses. Skin biopsy confirmed the diagnosis with irregular epidermal hyperplasia, wedge-shaped hypergranulosis, and a dense band-like lymphocytic infiltrate. This case demonstrates the full dermoscopic spectrum of HLP across lesions at different stages in a single patient at one time point, and highlights the probable role of chronic venous insufficiency in promoting Koebner-like lesion development. Our findings may assist clinicians in the non-invasive diagnosis and staging of HLP.

## Introduction

Lichen planus (LP) is a chronic immune-mediated inflammatory disorder affecting the skin, mucous membranes, and nails, with a worldwide prevalence of approximately 0.5–1% (1). Hypertrophic lichen planus (HLP) represents one of the most clinically challenging variants, characterized by thick, violaceous, verrucous plaques or nodules that are intensely pruritic and predominantly located on the lower extremities, particularly the pretibial regions and ankles (2). The pathogenesis of HLP involves autoreactive CD8 + T-cell-mediated cytotoxic attack on keratinocytes, leading to progressive epidermal hyperplasia, hypergranulosis, and dyskeratosis (2).

Dermoscopy has significantly advanced the non-invasive diagnosis of inflammatory skin diseases—a practice increasingly termed “inflammmoscopy” (3, 4). In classical LP, the pathognomonic dermoscopic feature is Wickham striae, appearing as white-to-yellowish reticular or annular structures on a reddish-violaceous background, often accompanied by dotted or hairpin vessels at the periphery (5). In HLP, additional features arise with progressive keratinocyte proliferation, including keratinous plugs—also called “corn pearls”—round white-yellowish globular structures representing dilated infundibula filled with cornified material (6). When numerous corn pearls coalesce into lobulated hyperkeratotic masses, the resulting dermoscopic pattern has been described as the “bouquet of white roses” sign, proposed as a specific marker for advanced HLP (7).

Despite several reports documenting individual dermoscopic features of HLP, sequential multi-stage dermoscopic documentation demonstrating the full evolution of lesions within a single patient has rarely been published. Moreover, the potential influence of underlying varicose veins and chronic venous insufficiency (CVI) on the distribution and progression of HLP has received limited systematic attention. Herein, we report a case that uniquely captures the complete dermoscopic spectrum of HLP—from the earliest inflammatory stage to the fully developed hypertrophic stage—simultaneously observed across different anatomical sites within the same patient at the same clinical encounter, and discuss the underlying pathophysiological implications.

## Case description

**Figure 1:**
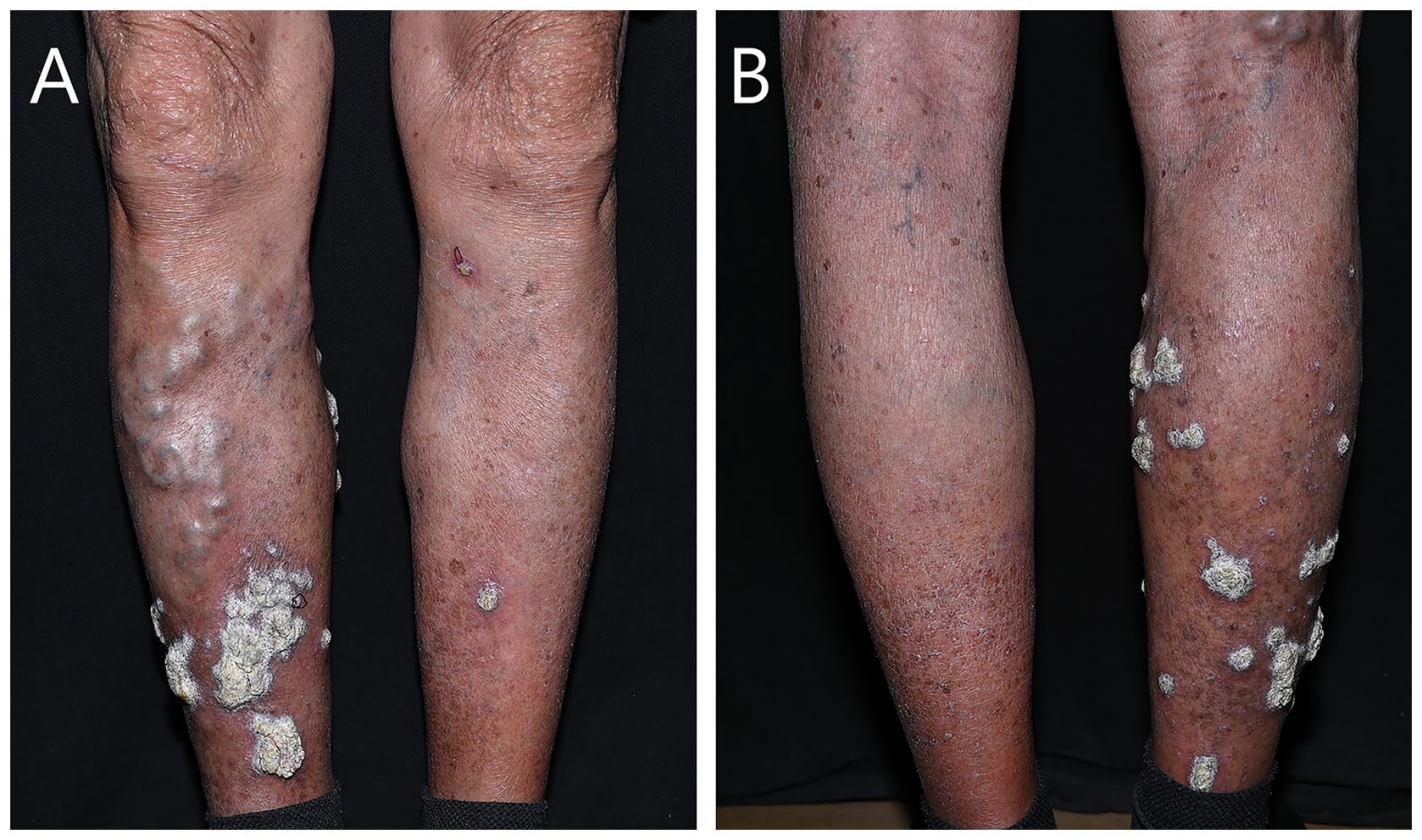
Multiple yellowish-brown verrucous hyperkeratotic papules and nodules confined to the lower leg, showing a striking ipsilateral distribution corresponding to the side affected by varicose veins. **(A)** Involvement on the extensor surface; **(B)** Involvement on the flexor surface.

A 62-year-old male presented to our dermatology outpatient department with a 6-month history of progressively increasing pruritic, hyperkeratotic nodules on the lower leg. He denied any recent drug intake or family history of similar skin conditions. Physical examination revealed prominent varicose veins predominantly affecting the right lower extremity. Multiple yellowish-brown, verrucous, hyperkeratotic papules and nodules of varying sizes were distributed on the lower leg, with a striking ipsilateral predilection for the side bearing the varicose veins (Figures 1A,B). The lesions were asymptomatic in terms of pain but associated with moderate pruritus. No oral mucosal, nail, or scalp involvement was identified.

## Diagnostic assessment

**Figure 2:**
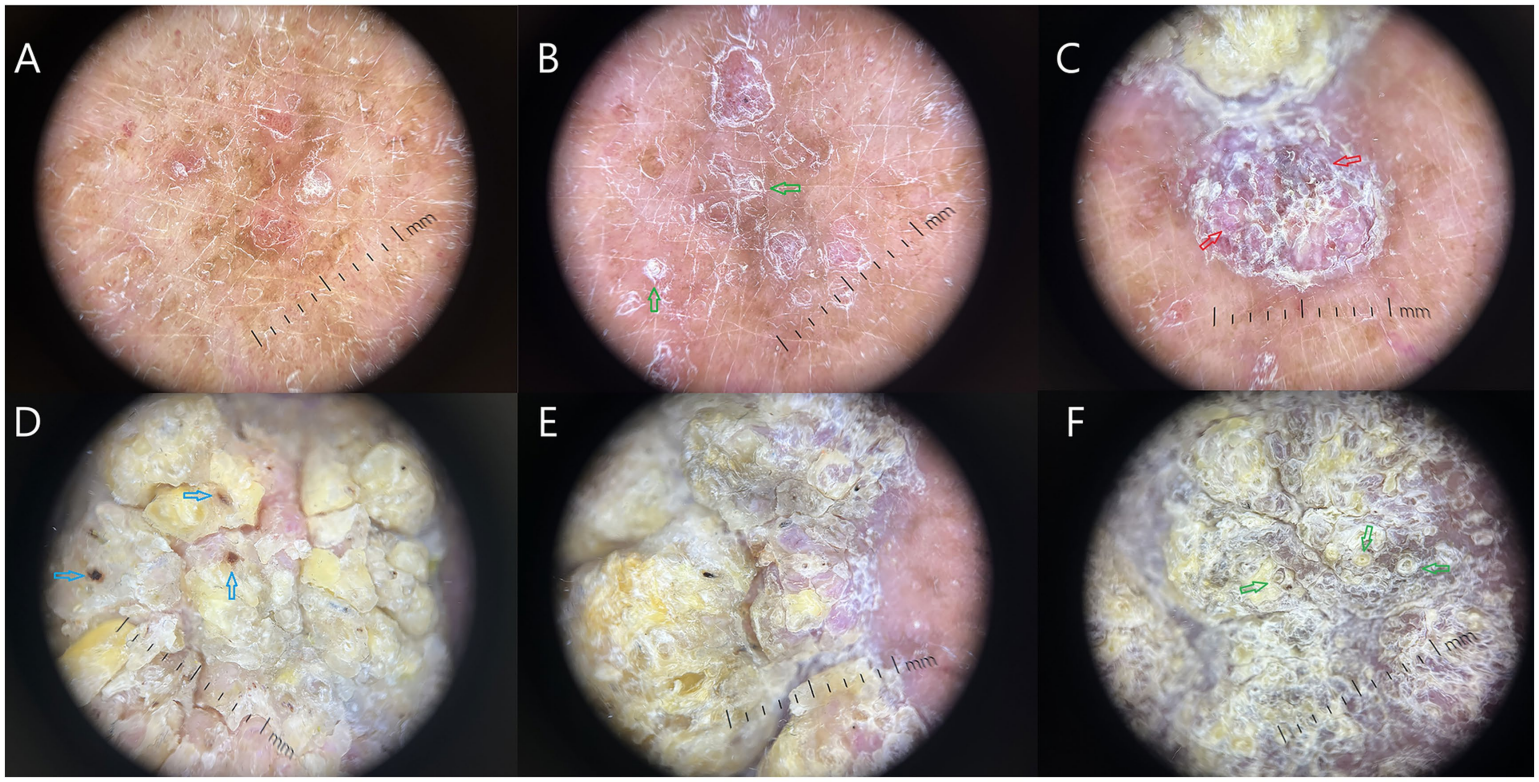
**(A)** Stage 1—minimally elevated lesion. Structureless pigmented areas with central hairpin and dotted vessels on an erythematous background. Peripheral white scales are visible. **(B)** Stage 2—moderately elevated lesion. Diffuse pigmentation with emerging Wickham striae (white reticular structures) and early corn pearls (green arrows). Vascular structures partially obscured by scaling. **(C)** Stage 3—markedly elevated, verrucous lesion. Elevated keratinous plugs with violaceous Wickham striae (red arrows) and yellow structureless areas: presenting “Volcano crater” appearance. **(D)** Stage 4a—fully developed lesion. Lobulated hyperkeratotic masses (“bouquet of white roses”) with part of reddish-brown dotted structures at lobule apices (blue arrows). Wickham striae are no longer visible. **(E)** Stage 4b—peripheral margin of a fully developed lesion. Stage 3-like dermoscopic features reappear at the margin, reflecting the centripetal-to-peripheral evolution of lesion maturation. **(F)** Stage 4c—alternative fully developed pattern. Dense compact lobulated hyperkeratotic masses with multiple keratinous plug aggregates (green arrows).

Dermoscopic examination was performed using a contact polarized-light dermatoscope (×10 magnification) on lesions at multiple distinct anatomical sites, revealing four evolutionarily distinct patterns:

Stage 1 (early lesion, minimally elevated): dermoscopy showed an erythematous-brownish background with structureless pigmented areas. In the central area encircled by pigmentation, hairpin-shaped and dotted vessels were evident. Focal areas of white scaling were noted peripherally (Figure 2A).
Stage 2 (moderately elevated lesion): the background pigmentation became more diffuse. The central vascular area was obscured and elevated with overlying white scales. Characteristically, reticular Wickham striae and early keratotic plugs (corn pearls) became identifiable (Figure 2B).
Stage 3 (markedly elevated, verrucous lesion): dermoscopy revealed prominently elevated keratinous plugs with a violaceous hue. Wickham striae remained partially visible in a lilac-violet coloration. Irregular yellowish structureless areas were interspersed among the keratinous masses. The overall appearance resembled a “volcano crater” morphology (Figure 2C).
Stage 4a (fully developed lesion — “bouquet of white roses”): the keratinous structures were markedly hypertrophied and coalesced into lobulated white-yellowish masses. Notably, reddish-brown dotted structures were visible at the apices of some lobules. Wickham striae were no longer identifiable, completely obscured by the overlying hyperkeratosis. This pattern corresponds to the characteristic “bouquet of white roses” sign (Figure 2D).
Stage 4b (peripheral margin of a fully developed lesion): at the periphery of the fully developed lesion, Stage 3-like features re-emerged, demonstrating a centripetal-to-peripheral dermoscopic gradient that reflects the sequential temporal evolution of lesion development from center to margin (Figure 2E).
Stage 4c (alternative fully developed pattern): another fully developed lesion displayed even denser, more compact lobulated keratinous structures with multiple keratinous plug aggregates. Wickham striae were entirely absent (Figure 2F).

**Figure 3:**
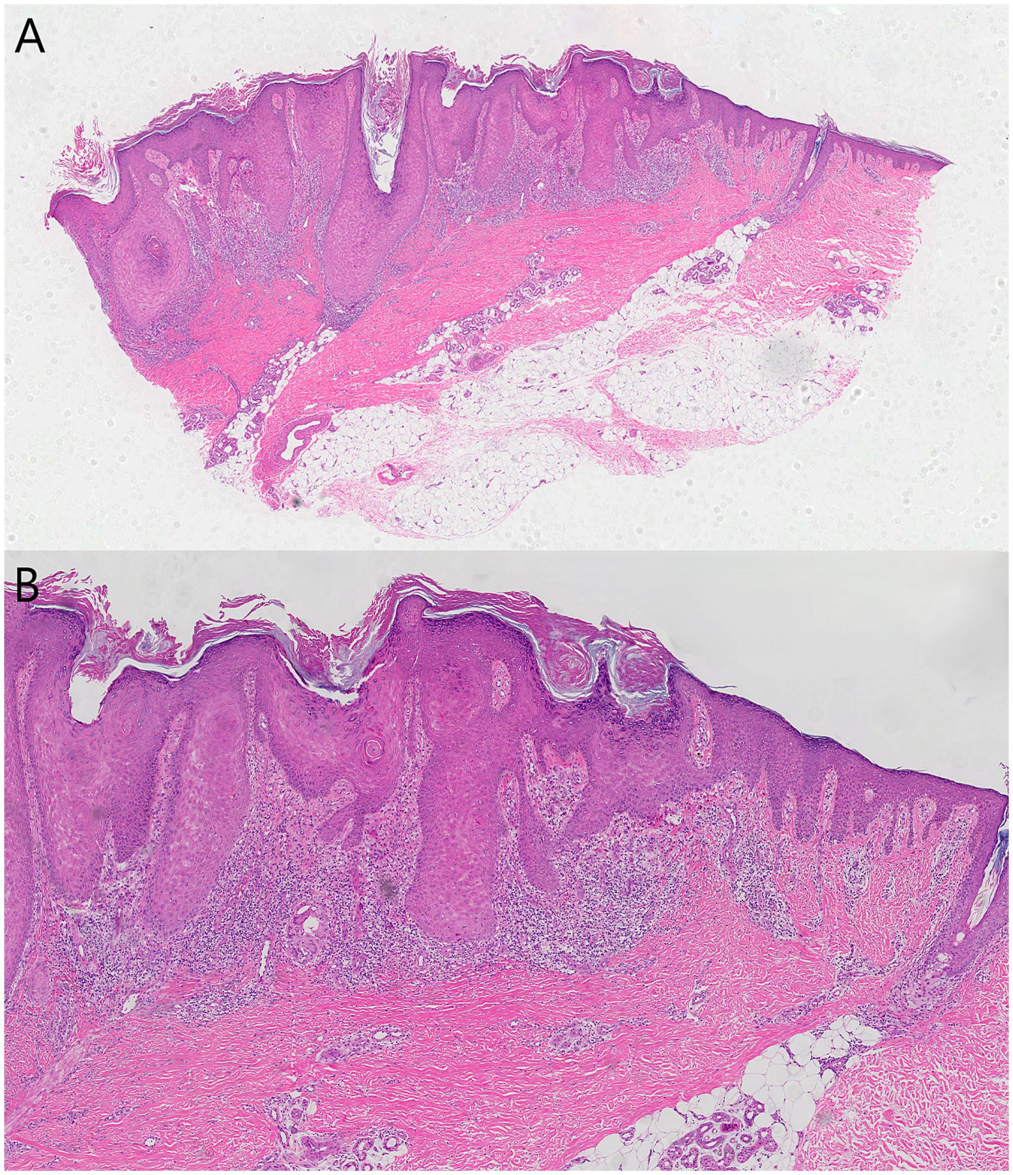
**(A)** Histopathological examination (hematoxylin & eosin, ×40) demonstrating pseudoepitheliomatous epidermal hyperplasia with prominent rete ridge elongation and orthohyperkeratosis. A follicular infundibular keratotic plug was observed. **(B)** A dense, band-like lichenoid lymphocytic infiltrate occupies the superficial dermis, with scattered apoptotic keratinocytes and basal cell vacuolation along the dermoepidermal junction.

A punch biopsy was obtained from a representative verrucous nodule. Histopathologically the epidermis showed pseudoepitheliomatous with prominent rete ridge elongation, accompanied by orthohyperkeratosis and conspicuous wedge-shaped hypergranulosis. A follicular infundibular keratotic plug was observed, characterized by a dilated follicular ostium filled with laminated cornified material with a central keratinous core, surrounded by a thickened perifollicular granular cell layer—a histopathological counterpart of the dermoscopic corn pearl (Figure 3A). A dense, band-like lichenoid lymphocytic infiltrate occupied the superficial dermis, and scattered apoptotic keratinocytes and basal cell vacuolation were present along the dermoepidermal junction (Figure 3B). These histopathological features in conjunction with the clinical and dermoscopic presentation confirmed the diagnosis of hypertrophic lichen planus.

## Discussion

LP is a chronic T-cell-mediated lichenoid dermatosis with multiple clinical variants. HLP is among the most severe cutaneous subtypes, clinically presenting with pruritic, hyperkeratotic, verrucous plaques typically on the lower legs. Compared with classical LP, HLP demonstrates more pronounced epidermal changes driven by chronic scratching, repetitive trauma, and progressive keratinocyte dysregulation (8). Histopathologically, HLP is defined by irregular acanthosis, wedge-shaped hypergranulosis, hyperkeratosis, and a dense lichenoid lymphocytic infiltrate—features confirmed in our case (2). Dermoscopy has proven invaluable in the non-invasive diagnosis of HLP. Wickham striae, the hallmark dermoscopic feature of LP, appear as white-yellowish reticular or annular structures reflecting the histological counterpart of wedge-shaped hypergranulosis (9). Errichetti and Stinco (4) demonstrated that dermoscopy (inflammoscopy) can reliably differentiate HLP from histopathologically similar conditions such as prurigo nodularis and hypertrophic lupus erythematosus, with the combination of Wickham striae and corn pearls offering high diagnostic specificity. The “bouquet of white roses” sign—coalescent lobulated corn pearls—was proposed by Malakar et al. (7) as a specific dermoscopic marker for advanced HLP, reflecting the maximal degree of epidermal hyperplasia and keratinization.

Our case uniquely documents an almost complete dermoscopic spectrum of HLP within a single patient by examining lesions at different anatomical sites at a single time point. We propose a four-stage dermoscopic model: Stage 1 corresponds to early inflammatory activity with structureless pigmented areas and visible hairpin/dotted vessels reflecting superficial vascular plexus involvement (2); Stage 2 reflects wedge-shaped hypergranulosis and blocked follicular infundibula, manifesting dermoscopically as emerging Wickham striae and early corn pearls (2); Stage 3 represents active verrucous transformation with a “volcano crater” morphology as keratinous plugs protrude, with Wickham striae still discernible (10); and Stage 4 represents fully developed HLP with the “bouquet of white roses” pattern, reflecting dense pseudoepitheliomatous acanthosis with overlying compact hyperkeratosis (7, 11). This dermoscopic progression parallels the histopathological continuum from early lichenoid inflammation to progressive pseudoepitheliomatous hyperplasia.

It is worth noting that we identified reddish-brown dotted structures at the apices of lobulated keratinous masses in Stage 4 lesions. Although similar structures have occasionally been found in previous publications, they have not received sufficient attention from the authors (6, 7). We propose that these reddish-brown dotted structures correspond to dilated, tortuous capillaries within dermal papillae that have been incorporated into the hypertrophied epidermal projections; their reddish-brown appearance likely reflects capillary microhemorrhage induced by scratching. Importantly, this feature may serve as a dermoscopic marker of Stage 4 HLP, helping to differentiate it from other hyperkeratotic nodular dermatoses such as prurigo nodularis.

In our patients, HLP lesions were more densely distributed on the lower limb with varicose veins. HLP shows a well-established predilection for the lower extremities, and several authors have speculated on the role of chronic venous insufficiency (CVI) in its pathogenesis (11–13). CVI causes sustained venous hypertension, resulting in extravasation of erythrocytes and plasma proteins, local hypoxia, oxidative stress, and a persistent pro-inflammatory microenvironment—all of which may create a more permissive substrate for accelerated lichenoid inflammation and progressive hyperkeratosis. These conditions may facilitate the Koebner phenomenon and provide a substrate for sustained CD8 + T-cell activation (11, 14). In this regard, the pathophysiological mechanism may be conceptually analogous to that of stasis dermatitis, in which venous hypertension-mediated perivascular fibrin cuffing, tissue hypoxia, and leukocyte extravasation perpetuate a chronic cutaneous inflammatory state (15). We hypothesize that this circulatory disparity between the two limbs accounts for the asymmetric coexistence of lesions at different dermoscopic stages within the same patient. While CVI-associated pathophysiological contributions have been mentioned in prior literature (11, 14, 15), this direct, visually compelling lateralization—combined with a dermoscopic stage gradient that mirrors the distribution of venous insufficiency—more strongly implicates circulatory disturbance as a lesion-promoting and lesion-advancing factor in HLP than previously appreciated.

Previous investigators have reported sequential dermoscopic changes within the same anatomical region of a single patient monitored over a period of several months (7). However, to our knowledge, such a comprehensive cross-sectional dermoscopic documentation—capturing coexisting lesions spanning nearly the full evolutionary spectrum of HLP at a single clinical encounter—has not been previously reported. This case therefore offers a “frozen moment” view of HLP’s natural dermoscopic history.

The main limitation of this study is that the diagnosis of varicose veins was based solely on dermatological physical examination and the patient’s self-reported history, without objective vascular imaging. The association between CVI and the asymmetric distribution of HLP is a clinical observation and reasonable hypothesis. Future examinations using systematic duplex ultrasound, transcutaneous oxygen pressure (tcPO2), and skin perfusion pressure would be needed to better characterize the hemodynamic parameters that may promote HLP development.

## Conclusion

We report a case of HLP with a unique distribution pattern strongly associated with ipsilateral varicose veins and demonstrating the complete dermoscopic evolutionary spectrum across four sequential stages within a single patient. This case highlights the diagnostic and prognostic value of dermoscopy in HLP and underscores the importance of vascular comorbidity assessment in patients with lower extremity HLP.

## Data Availability

The original contributions presented in the study are included in the article/supplementary material, further inquiries can be directed to the corresponding author.
